# *MHO1,* an Evolutionarily Conserved Gene, Is Synthetic Lethal with *PLC1;* Mho1p Has a Role in Invasive Growth

**DOI:** 10.1371/journal.pone.0032501

**Published:** 2012-03-07

**Authors:** Ivan D. Schlatter, Maria Meira, Vanessa Ueberschlag, Dominic Hoepfner, Rao Movva, Nancy E. Hynes

**Affiliations:** 1 Friedrich Miescher Institute for Biomedical Research, Basel, Switzerland; 2 Novartis Institutes for Biomedical Research, Basel, Switzerland; Université de Nice-CNRS, France

## Abstract

The novel protein Memo (Mediator of ErbB2 driven cell motility) was identified in a screen for ErbB2 interacting proteins and found to have an essential function in cell motility. Memo is evolutionarily conserved with homologs found in all branches of life; the human and yeast proteins have a similarity of >50%. In the present study we used the model organism *S. cerevisiae* to characterize the Memo-homologue Mho1 (Yjr008wp) and to investigate its function in yeast. In a synthetic lethal screen we found *MHO1* as a novel synthetic lethal partner of *PLC1,* which encodes the single phospholipase C in yeast. Double-deleted cells lacking *MHO1* and *PLC1,* proliferate for up to ten generations. Introduction of human Memo into the *memoΔplc1Δ* strain rescued the synthetic lethal phenotype suggesting that yeast and human proteins have similar functions. Mho1 is present in the cytoplasm and the nucleus of yeast cells; the same distribution of Memo was found in mammalian cells. None of the Memo homologues have a characteristic nuclear localization sequence, however, a conserved nuclear export sequence is found in all. In mammalian cells, blocking nuclear export with Leptomycin B led to nuclear Memo accumulation, suggesting that it is actively exported from the nucleus. In yeast *MHO1* expression is induced by stress conditions. Since invasive growth in *S. cerevisiea* is also stress-induced, we tested Mho1's role in this response. *MHO1* deletion had no effect on invasion induced by nutrient deprivation, however, Mho1 overexpression blocked the invasive ability of yeast cells, suggesting that Mho1 might be acting in a dominant negative manner. Taken together, our results show that *MHO1* is a novel synthetic lethal interactor with *PLC1*, and that both gene products are required for proliferation. Moreover, a role for Memo in cell motility/invasion appears to be conserved across species.

## Introduction

Memo (Mediator of ErbB2 driven cell motility) was identified in a screen for ErbB2 receptor tyrosine kinase (RTK) interacting proteins with roles in cell motility. Memo specifically interacted with a phospho-peptide encompassing an autophosphorylation site in the C-terminus of ErbB2. Treatment of tumor cells with the ErbB2 activating ligand heregulin (HRG) induces migration and in siRNA-mediated Memo knock-down (KD) cells, HRG-induced migration is dramatically impaired [1], [2].

Memo is encoded by a gene found in all kingdoms of life, and multiple sequence alignment showed that the protein sequence is highly conserved. Memo has no obvious functional domain and does not belong to a known protein family. Interestingly, Memo is structurally homologous to a LigB, a class III non-heme iron binding extradiol dioxygenases [3]. While tempting to speculate that Memo might have enzymatic activity, the catalytic activity of LigB family members is mediated by an iron ion, which is coordinated by two His residues and a Glu residue. In the Memo family, the Glu residue has been replaced by a Cys residue, making it unlikely to have the same activity as the bacterial enzyme [3]. The yeast gene *YJR008W* shows more than 50% similarity with the human gene and was named *MHO1* (Memo HOmolog 1). In the present study, we investigated the function of Mho1 (Yjr008wp) in *S. cerevisiae.* We show here that *MHO1* is a novel synthetic lethal (SL) interactor with *PLC1.* Yeast cells germinate in the absence of both genes, however, they cannot proliferate. Moreover, we found that under conditions of nutrient insufficiency Mho1 plays a role in haploid invasive growth. This suggests that a function for Memo in cell motility/invasion is conserved across species.

## Results

### *MEMO* is a single copy gene conserved throughout evolution

**Figure 1:**
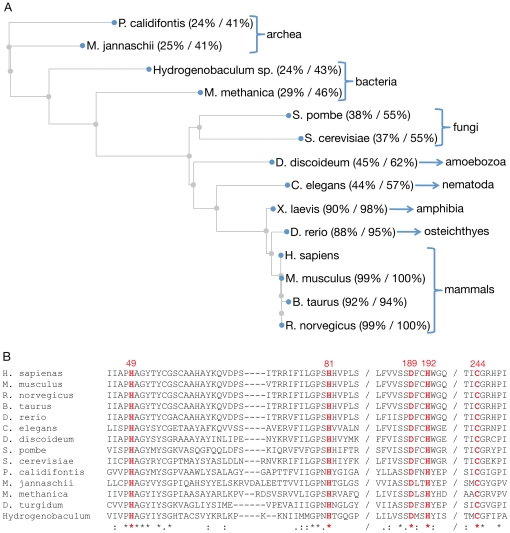
Phylogenetic tree and sequence alignment of Memo homologues in all kingdoms of life. (A) The evolutionary distances between Memo protein sequences in the listed species are shown, using the human Memo as the query sequence. The % identity and % similarity, respectively, are indicated in brackets. (B) The protein sequence of Memo homologues in the species shown in panel A are presented. The red letters indicate the conserved amino acids in the putative “active site” [3].

To identify *MEMO* homologs in other sequenced organisms, we performed a blastp search (Basic Local Alignment Search Tool), using the blastp tool provided from NCBI (as described in [4]), and the human Memo protein sequence as query. One copy of a *MEMO* homolog was found in various species representing all kingdoms of life (Figure 1A). By performing multiple sequence alignments we could show that Memo is highly conserved. The human and the yeast protein share an identity of more than 40% and a similarity of more than 50%. Some amino acids are completely conserved from bacteria to human; in particular those close to the predicted “vestigial active site” of Memo (indicated in red, Figure 1B). This suggests that these amino acids could be essential, for example, for a potential enzymatic activity, or as part of a novel conserved binding domain. The yeast gene, *YJR008W,* was named *MHO1* (Memo HOmolog 1). In this paper the human gene and protein are referred to as *MEMO* and Memo, respectively; the yeast gene and protein are referred to as *MHO1* and Mho1, respectively.

### Examination of effects of *MHO1* deletion in *S. cerevisae*

*MHO1* was deleted in the wild-type strains BY4741, BY4742, and BY4743 [5] by standard methods. The deletion of *MHO1* in haploid or diploid cells had no effect on viability, nor did it affect growth in liquid culture at 20°C, 30°C, and 37°C. The *mho1Δ* cells were also tested for their sensitivity to various agents including hydroxyurea (HU), NaCl, CoCl_2_, rapamycin, and benomyl. No difference in growth was observed in *mho1Δ* cells compared to the wild-type cells (Figure S1).

**Figure 2:**
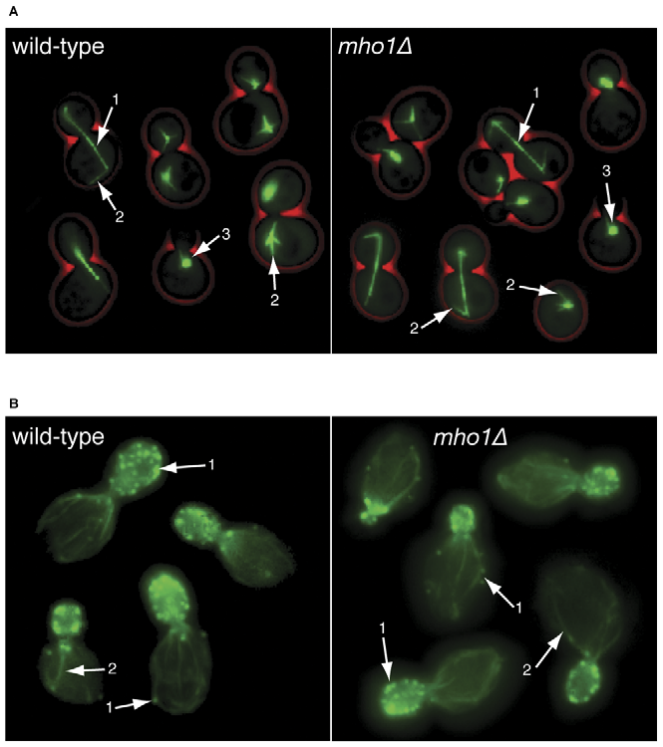
Cytoskeleton analysis of wild-type and *memoΔ* strains of *S. cerevisiae.* (A) To stain the tubulin cytoskeleton, a GFP-tagged TUB1 expression vector, under its endogenous promoter was introduced into wild type and *memoΔ* cells. The arrows indicate (1) nuclear microtubules, (2) astral microtubules, and (3) the spindle pole body. (B) Phalloidin-OregonGreen was used to stain wild type and *memoΔ* cells. Two actin structures are stained: (1) cortical actin patches and (2) actin cables. There are no obvious differences in the microtubule structures or the actin cytoskeleton comparing wild-type and *memoΔ* cells.

Memo is required for migration of tumor cells in response to activation of various RTKs and Memo KD impacts on the actin and microtubule (MT) cytoskeleton [1], [2]. Accordingly, we investigated if Mho1 has a role in yeast motility and cytoskeletal morphology. To visualize the MTs, we used a yeast strain expressing a copy of *TUB1-GFP* using the plasmid pAFS125 [6]. *MHO1* deletion was introduced into this strain by replacement with the *klTRP1* selection marker. Using fluorescent microscopy, we observed no difference in the microtubule cytoskeleton between wild-type and *mho1*Δ strains (Figure 2A; 1 and 2, nuclear and astral microtubules, respectively; 3, spindle pole body). The actin-containing structures (reviewed in [7]) were also examined by microscopy. Neither the cortical actin patches, nor the actin cables differed between the wild-type and *mho1Δ* strains (Figure 2B, 1 and 2, respectively,); actin rings or actin at the site of polarized growth were also unaffected by Mho1 loss. Thus, Mho1 is not required to maintain normal actin or MT cytoskeletal structures.

### The deletion of *MHO1* in fungal species does not affect polarized growth

**Figure 3:**
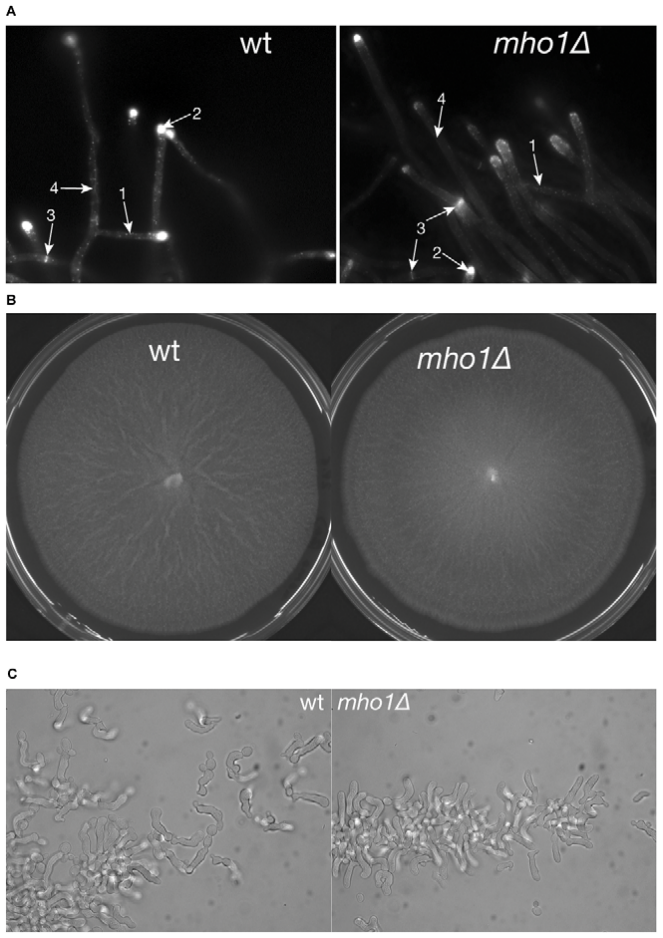
Examination of filamentous growth in wild type and *memoΔ* cells. (A) Actin was visualized in wild type and *memoΔ Ashbya gossypii* by staining with Phalloidin-OregonGreen. The stained actin structures are: 1) cortical actin patches, 2) actin patches at the tip, 3) actin rings at sites of septation, and 4) actin cables. There are no observable differences in the actin cytoskeleton between wild type and *memoΔ* strains. (B) A polarized growth assay was performed in *Ashbya gossypii*. Spores from the wild type and the *memoΔ* strain were spotted in the middle of an agar plate with full medium and radial growth was measured during 7 days. The picture was taken at the end of the experiment. (C) Shmoo formation of wild type and *memoΔ* cells was visualized on YPD agar-coated microscopy slides, following stimulation of MATa cells for 4 hrs with α-factor.

To investigate if Mho1 has a role in polarized growth in fungi, we used the well-studied model organism *Asbya gossypii* [8], a filamentous growing ascomycete. Since filaments can grow at 200 µm/h, *A. gossypii* requires sustained polarisation at the growing tip; crucial is the interplay between the actin cytoskeleton, microtubules, and proteins involved in polarised growth [9], [10]. The *A. gossypii* homolog gene is *AGR321W,* which we will refer to as *MHO1,* for simplicity. The *A. gossypii MHO1* deletion strain was generated by standard means [11]. The *mho1Δ* strain was viable and sporulation efficiency and spore morphology were identical to the wild-type strain. Four actin structures are well defined in *A. gossypii*: 1) cortical actin patches along the entire hypha, 2) actin patches at the tip, 3) actin rings at the site of septation, and 4) actin cables. In Phalloidin-FITC labelled cells, all four structures were indistinguishable between the wild-type and the *mho1Δ* strain (1–4, respectively, in Figure 3A). We also measured radial growth speed after inoculating a few spores on an agar Petri dish containing full medium. Both wild-type and *mho1Δ* strains covered the entire plate within seven days (Figure 3B). No differences in growth speed, hyphal morphology or branching patterns were observed.

*A. gossypii* always uses filamentous growth; there is no switch from isotropic to polarized growth in response to external stimuli or changes in growth conditions. Thus, we also investigated the role of Mho1 in the *S. cerevisiae* mating response. In order to mate, haploid cells recognise pheromone of the opposite mating type cell and extend a shmoo towards the pheromone gradient (reviewed in [12]). Deletion of *MHO1* had no effect on the elongation of a “hyphal-like” structure in *S. cerevisiae* (Figure 3C) and mating efficiency was similar to wild-type.

### Mho1 is localized to the nucleus and the cytoplasm

**Figure 4:**
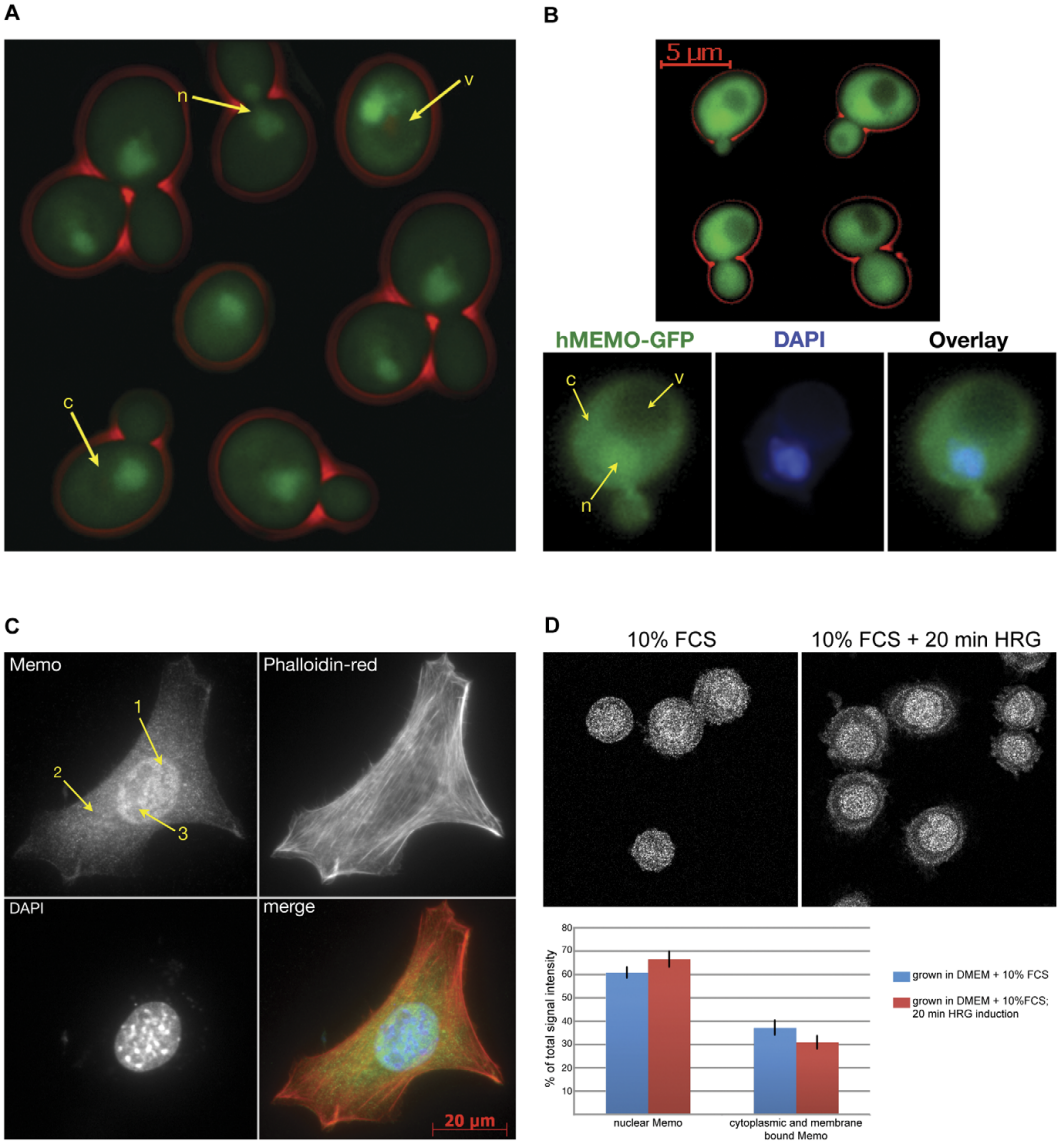
Cellular localization of Memo in yeast and mammalian cells. (A) Endogenous Mho1 was tagged C-terminally with GFP by homologous recombination for visualization. Yeast cells were grown 48 hrs to stationary phase in YPD and GFP was visualised by fluorescence microscopy. Mho1-GFP is present in the cytoplasm (c), the nucleus (n), and is excluded from the vacuole (v). (B) The human Memo-GFP was expressed in yeast cells and visualized. Memo is present in the cytoplasm, the nucleus and excluded from the vacuole. (C) Endogenous Memo was visualized in mouse embryonic fibroblasts (MEFs) using a specific Memo antiserum [1]. Memo is present in the cytoplasm (2), the nucleus (1) and is excluded from the nucleoli (3). (D) The same antibody was used for Memo staining in SKBR3 breast tumor cells. (left panel) Memo is found in the cytoplasm and the nucleus in cells grown in DMEM containing 10% FCS. (right panel) following treatment of cultures for 20 min with 10 nM HRG, Memo is recruited to the membrane. Quantification of nuclear signal intensity versus signal intensity outside the nucleus showed that the levels of nuclear Memo are approximately the same before and after the treatment.

To follow Mho1 sub-cellular localization, the endogenous protein was tagged at its C-terminus with GFP, by standard methods [13]. Mho1-GFP expressing cells were grown to stationary phase, since Mho1 expression is up-regulated under these conditions (Figures S2 and S3), and its localization was examined by fluorescent microscopy. Mho1-GFP was found in the cytoplasm and the nucleus; no GFP signal was detected in the vacuole (Figure 4A, labelled c, n and v, resp.). Although Mho1 expression levels are lower in log-phase cells, a similar distribution was observed. To examine the localization of the human Memo in yeast, we cloned the GFP-tagged hMEMO gene into an integrative plasmid, which was integrated into the Trp1 locus. hMEMO expression was induced by galactose and GFP was visualized. Human Memo-GFP has a similar distribution as the yeast protein, and is present in cytoplasm and nucleus, but not in the vacuole (Figure 4B).

Memo localisation was also examined by immunofluorescence (IF) in mammalian cells using a Memo-specific specific antibody [1]. IF for Memo carried out on mouse embryonic fibroblasts (MEFs) revealed cytoplasmic and nuclear staining, with exclusion from the nucleoli (Figure 4C). IF for Memo in SKBR3 breast tumor cells revealed that in growth medium containing 10% fetal calf serum, Memo was distributed in the cytoplasm and the nucleus (Figure 4D, left panel). In response to ErbB2 activation, in HRG-treated cultures, the cytoplasmic Memo is strongly localized at the plasma membrane and appears enhanced in the nucleus (Figure 4D, right panel). However, quantification of the images did not reveal any significant differences in nuclear Memo in the two conditions. Thus, the human and the yeast protein are localized to similar sub-cellular compartments.

### Memo is actively exported from the nucleus

**Figure 5:**
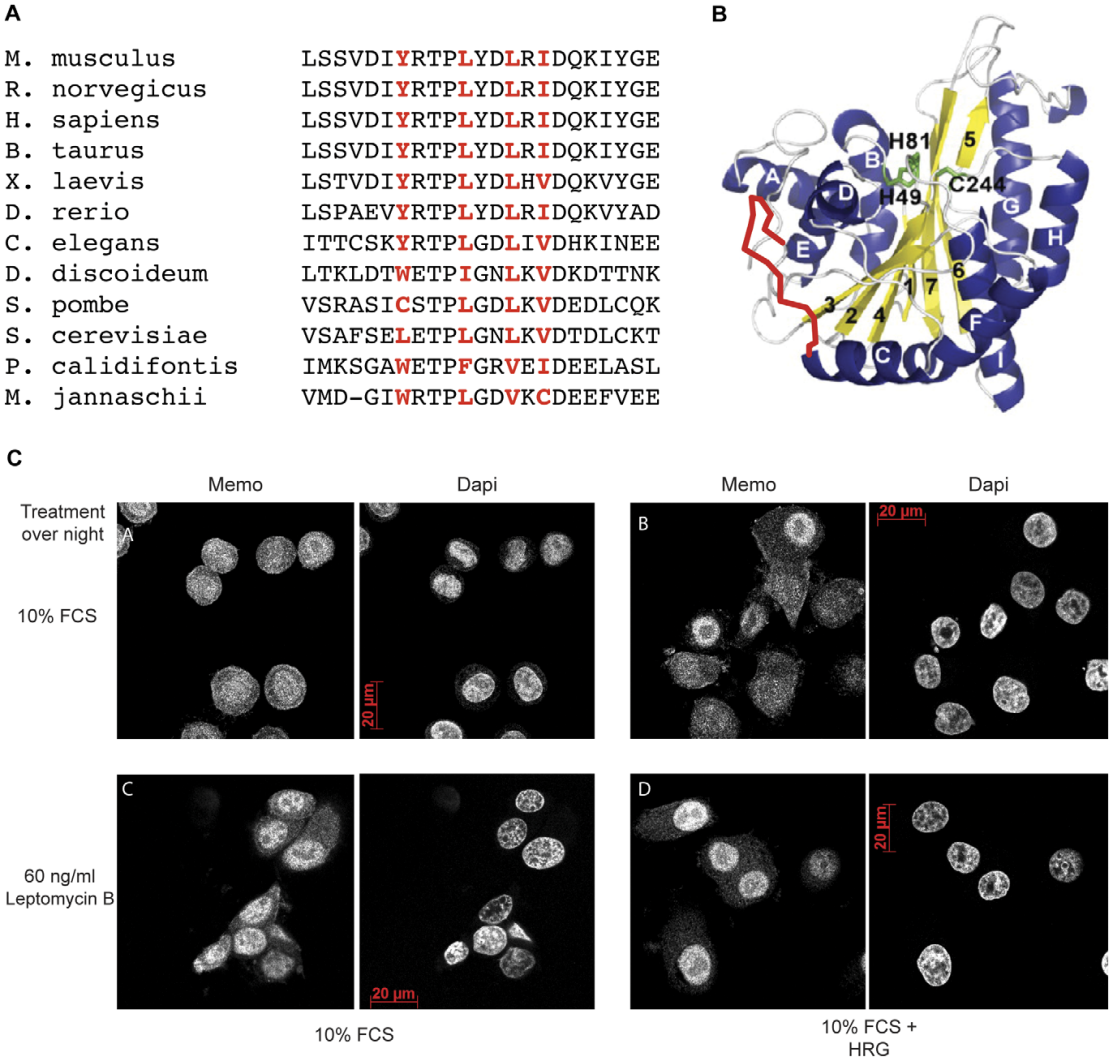
Identification of a functional nuclear export sequence in Memo homologues. (A) Using the NetNES 1.1 nuclear export sequence prediction software from CBS (Center for Biological Sequence analysis) a predicted NES, which is conserved in all examined species, was identified and highlighted in red. L_xxx_L_xx_L_x_L (L = Leucine or hydrophobic amino acid and _X_ = any amino acid). (B) The NES of Memo is located between β-sheet “2” and α-helix“C” on a 15 amino acid loop on the surface of the protein (indicated by the red line). (C) IF for endogenous Memo in human SKBR3 breast tumor cells. The cells were treated with: 10% FCS −/+ Leptomycin B (panels A vs C), 10% FCS+HRG −/+ Leptomycin B (panels B vs D). Leptomycin B treatment was overnight. Dapi stained nuclei are shown to the right of each panel.

Although Memo and Mho1 were present in the nucleus of yeast and mammalian cells, neither protein has a nuclear localization signal (NLS). However, a consensus nuclear export sequence (NES), L_xxx_L_xx_L_x_L (L = Leucine or hydrophobic amino acid and _X_ = any amino acid), was identified in both proteins and was found to be conserved throughout evolution (Figure 5A). Using the 3D protein structure of Memo [3] as a model, the NES is located between β-sheet “2” and α-helix“C” on an approximately 15 residue loop on the surface of the protein (red ribbon in Figure 5B).

The 33 KDa Memo could diffuse through the nuclear pore, but might also be actively imported and/or exported from the nucleus. To test this, we used SKBR3 human breast tumor cells and examined the effects of HRG and the nuclear export inhibitor Leptomycin B [14] on Memo distribution. IF revealed that there was no significant difference between nuclear Memo levels in the control compared to the HRG treated cultures (Figure 5C, panel A vs. B), confirming the results shown in Figure 4D. To block export, SKBR3 cultures were treated overnight with 60 ng/ml Leptomycin B, then were either left untreated or were exposed shortly to HRG. Compared to the control cells in 10% FCS, there was a significant accumulation of nuclear Memo in the Leptomycin B treated SKBR3 tumor cells (Figure 5C, panel A vs C). Moreover, after 20 min of HRG treatment, there was a further increase in nuclear Memo in the Leptomycin B treated cultures (Figure 5C, panel C vs D). These results suggest that Memo might enter the nucleus passively, either alone or in a complex, but once in the nucleus Memo is actively exported.

### *MHO1* is synthetic lethal with *PLC1*

**Figure 6:**
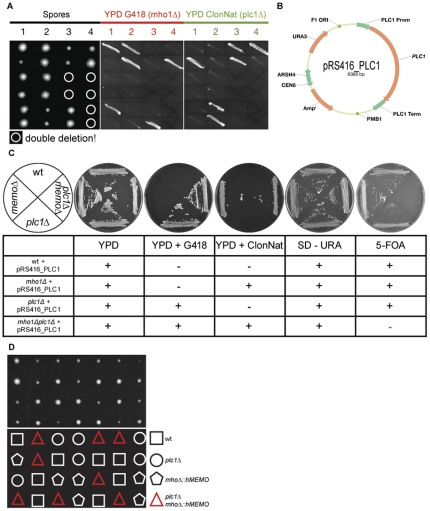
*MEMO* is synthetic lethal with *PLC1*. (A) A *memoΔ* a-strain (memo::kanMX) was mated to a *plc1Δ* α-strain (plc1::natMX). The resulting diploid strain was sporulated, the spores were grown on YPD plates and growing colonies were streaked out on YPD plates with G418 or ClonNat to test for *memoΔ* and *plc1Δ*, respectively. The location of the *memoΔplc1Δ* spores, where there was no growth, is indicated by white circles. *plc1Δ* colonies grow slower and are smaller. (B) The *PLC1* gene including its endogenous promoter and terminator was cloned into a CEN/ARS plasmid with the *URA3* selection marker (pRS416_*PLC1*). (C) By sporulating a diploid heterozygous *mho1Δ/MHO1; plc1Δ/PLC1* deletion strain harboring the *PLC1* expressing CEN/ARS plasmid, the three haploid deletion strains: *memoΔ*, *plc1Δ*, and *memoΔ plc1Δ* were created. These three and the wild-type strain were grown on SD-URA plates to select for the pRS416_*PLC1* plasmid. When streaked on a 5-FOA plate, which selects for loss of the plasmid, all but the double-deleted cells grow, showing that once Plc1 expression is lost, cells stop growing. (D) A *mho1::hMEMO_natMX* strain was mated with a *plc1::kanMX* strain. The resulting diploid strain was sporulated and the spores were dissected on YPD. The upper portion of the figure shows the growing colonies, the lower portion shows the phenotype. The red triangles indicate the *mho1::hMEMO_natMX plc1::kanMX* strain proving that the human protein can complement for Mho1 in this assay.

To further analyze Mho1 function, we performed a SL screen in *S. cerevisiae.* A *MHO1* deleted strain was made and mated with each of the 4800 viable haploid deletion strains. The only strain that showed a SL phenotype with *MHO1* deletion was *PLC1* deletion, the gene encoding the single isoform of phospholipase C found in yeast. To verify these results, we examined the growth of the wild-type strain and strains individually and double- deleted in *MHO1* or *PLC1.* This was accomplished by constructing Matα *mho1::natMX* and Mata *plc1::kanMX* strains, which were mated and sporulated. The dissected spores were then tested for growth on YPD plates plus G418 or ClonNat. Only wild-type cells, and *mho1Δ* or *plc1Δ* deleted cells could grow; none of the double-deleted cells grew (highlighted in Figure 6A with a white circle). It should be mentioned that the *plc1Δ* colonies are smaller since the deleted cells grow more slowly. In summary, the SL interaction between *MHO1* and *PLC1* uncovered in the large scale screen, could be verified.

Next, we analysed which stage of the yeast life cycle shows the SL phenotype. For this, we cloned the *PLC1* gene including its endogenous promoter and terminator in a CEN/ARS plasmid with the *URA3* selection marker (Figure 6B), and introduced it into the *plc1Δ* cells. After mating with the *mho1Δ* strain and subsequent sporulation, the wild-type, *mho1Δ*, *plc1Δ*, and *mho1Δ plc1Δ* stains, each with the pRS416_PLC1 plasmid, were isolated. After plating on 5-Fluoroorotic acid (5-FOA), which selects for loss of the *URA3* marker, all but the double-deleted strain grew (Figure 6C). Once the double-deleted cells lose the Plc1p expression plasmid, the cells stop proliferating. The results show that the phenotype is not at the germination level since spores do germinate and undergo up to ten cell divisions. Thus, the experiment shows that Mho1 and Plc1 are not required for germination, but are needed for proliferation.

### Human *MEMO* can replace *MHO1* and rescue the SL phenotype with the *plc1Δ* strain

The yeast and the human gene share a similarity of >50% (Figure 1A). We tested if human *MEMO* can complement the function of the yeast gene in *plc1Δ* cells by replacing the yeast gene with the human gene. By mating the *mho1::hMEMO_natMX* strain with a *plc1::kanMX* strain and dissecting spores, we could show that haploid strains carrying both selection markers grew. These results demonstrate that human Memo can replace the yeast protein and rescue the SL phenotype with *plc1Δ* (Figure 6D).

### Mho1 overexpression blocks haploid invasive growth

**Figure 7:**
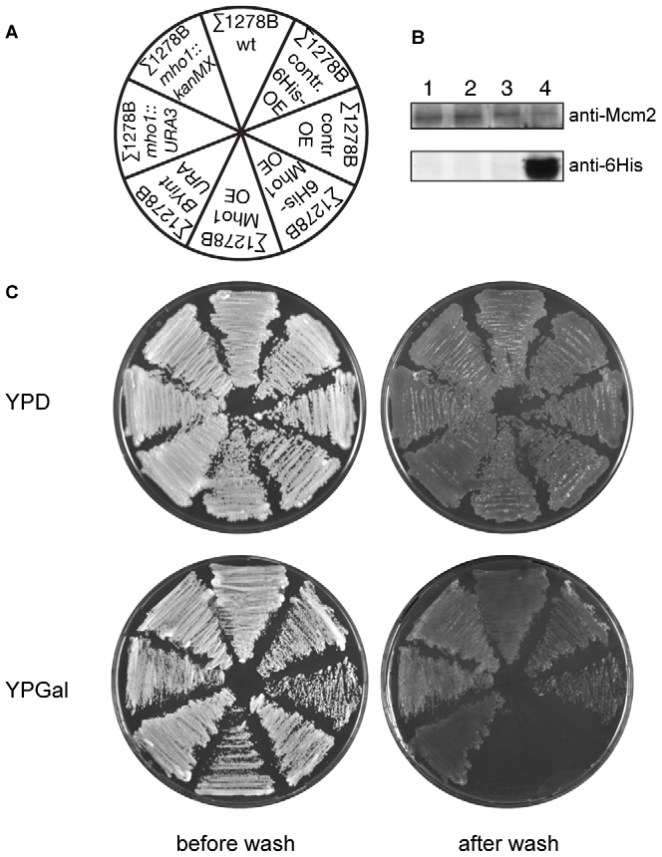
Overexpression of Mho1 abolishes invasive growth in the haploid Σ1278B strain. (A) Mho1 was overexpressed in the invasive yeast strain Σ1278B by integrating the pRS416_pGAL_*6HIS-MHO1* and the BYintURA_pGAL_*MHO1* plasmids. The *mho1Δ* strains were made by replacing the endogenous gene with *kanMX* or *URA3MX*. (B) Overexpression of 6His-Mho1 is shown by western blotting using a His-tag specific antiserum. Mcm2 levels were used as a loading control. The lysates were: 1) Σ1278B wt grown on YPD, 2) Σ1278B wt grown on YPGal, 3) Σ1278B 6His-Mho1 OE grown on YPD, and 4) Σ1278B 6His-Mho1 OE grown on YPGal. (C) To test for invasive growth potential in Mho1-lacking or overexpressing strains, the indicated strains were streaked out on YPD or YPGal (overexpressing conditions) agar plates. As controls, the wt Σ1278B or Σ1278B harbouring the empty pRS416_pGAL and BYintURA_pGAL plasmid were used. After four days, the plates were washed under the water tap. Only cells overexpressing Mho1 on the YPGal plates were washed off the plates, showing that overexpression blocks haploid invasive growth.

A large-scale screen examining the effects of gene disruption and overexpression on alcohol-induced filamentous growth uncovered Mho1 as having a potential function in this stress-induced process [15]. Not only alcohol, but other growth conditions including nutrient deprivation induce filamentous growth [16]. Under these conditions a haploid strain extends invasive filaments downward, which is referred to as haploid invasive growth [17]. Thus, we tested if Mho1 expression levels influenced haploid invasive growth in conditions of nutrient insufficiency. For this, Mho1 overexpression and deletion strains were made in the haploid invasive wild-type yeast strain Σ1278B [18]. Two deletion strains (Σ1278B *mho1*::kanMX and Σ1278B *mho1::*URA3) and two overexpression strains (Σ1278B *MHO1::OE* and Σ1278B *His-MHO1::OE*) were constructed (Figure 7A). To verify overexpression in response to galactose, extracts from Σ1278B *His-MHO1::OE* were tested in a western analysis using a His specific antiserum to detect Mho1. Only the extracts from cells grown on galactose showed high levels of His-Mho1 (Figure 7B).

To monitor invasive growth, the deletion and overexpression strains as well as control strains were streaked on YPD or YPGal plates, incubated for four days, and then washed with tap water to assess invasive potential. The deletion of Mho1 did not influence the invasion of the cells into the agar in any of the growth conditions (Figure 7C). In contrast, on the Gal plates, the two strains overexpressing Mho1 (Σ1278B *MHO1::OE* and Σ1278B *His-MHO1::OE*) failed to invade (Figure 7C). Taken together, the results suggest that while Mho1 is not essential for invasive growth, abnormal levels of the protein impede the process.

## Discussion

Memo is evolutionarily conserved with homologs found in all branches of life. Memo was initially identified based on its importance in breast cancer cell motility in response to ErbB2 activation [2], where it was shown to have an essential role in promoting the directionality of motile cancer cells [1]. The human and yeast proteins have a similarity of >50% and in the work presented here we used *S. cerevisiae* to characterize Mho1, the yeast homolog. We uncovered a role for Mho1 in haploid invasive growth, which might be linked to its function in mammalian cells, suggesting that this activity is conserved across species. Moreover, we uncovered a novel function for Mho1, namely a synthetic lethal interaction with *PLC1.* To date the genes that have been identified as SL with *PLC1,* that is *BUB1, BUB3* and *CBF1,* all have roles in spindle-assembly checkpoint and damage (Table S1). Mho1 has not been implicated in these processes. Nonetheless we tested if there might be a SL interaction between *mho1Δ* and these genes. All the double-deleted strains grew normally. Moreover, based on Plc1's role in the cAMP/PKA pathway we also tested nine non-essential genes involved with this pathway for a SL interaction with *mho1Δ* (Table S1). All the double deleted strains grew normally.

Based on the importance of Memo in mammalian cell motility, we expected to find a role for Mho1 in yeast MT and/or actin dynamics. However, we were unable to uncover any differences between *mho1Δ* cells and wild-type cells. Neither the MTs nor the actin-containing structures in proliferating *S. cerevisiae* were abnormal in *mho1Δ* cells. Moreover, the filamentous ascomycete *A. gossypii,* which requires coordinated interactions between the actin and the MT cytoskeleton for growth, was not affected by *MHO1* deletion. However, we noticed during our studies that Mho1 levels increased as cells reached stationary phase. A search of publicly available data bases, revealed that *MHO1* RNA expression is induced 5-fold or more in stationary phase and other stress conditions [19] (Figures S2 and S3). While *mho1Δ* cells behaved similarly to wild-type cells in many of the stress conditions, we found that nutrient-deprived stress-induced haploid invasive growth was blocked in the presence of high Mho1 levels. Since *mho1Δ* cells were not impaired in haploid invasive growth, these results suggest that overexpressed Mho1p behaves in a dominant negative fashion. In summary, the results suggest that Mho1 might not have an essential role in normal cellular situations, but in a stress condition related to migration/invasion Mho1 levels need to be maintained at physiological levels for a normal response.

A large set of genes (approximately 900) showed a similar response to most of the environmental changes that induce *MHO1* expression [19]. Promoter analysis and subsequent characterization of the responses of strains mutant for some of these genes implicated the transcription factors Yap1, Msn2p and Msn4p in mediating the transcriptional response [19]. We analysed the promoter region of *MHO1* to find binding sites for transcription factors and uncovered potential sites for Msn2/Msn4 and Ino2/Ino4 (Figure S4A). *MSN4* gene expression is itself Msn2/4p dependent and induced by stress, while *MSN2* expression is constitutive [19]. By consulting the data base from YEASTRACT (Yeast Search for Transcriptional Regulators And Consensus Tracking) [20] we found additional TF that could potentially directly or indirectly affect *MHO1* transcription (Figure S4B).

In the SL screen we found *MHO1* as a novel SL partner of *PLC1.* Introduction of human Memo into the *memoΔplc1Δ* strain rescued the SL phenotype suggesting that yeast and human proteins have similar functions. The mechanism underlying the SL interaction is not currently known, however, it is interesting to discuss some possibilities. Plc1p hydrolyzes the membrane phospholipid PtdIns(4,5)P2 to produce 1,2 diacylglycerol (DAG) and inositol 1,4,5-trisphosphate (IP3). In mammalian cells, DAG is needed for activation of some PKC isoforms. In yeast, C1, the potential DAG binding domain of Pkcp, is not required to restore viability to pkc*Δ* cells, suggesting that Pkcp is not involved in the SL phenotype. Moreover, in yeast multiple pathways provide DAG, making it unlikely that low DAG levels are responsible for the SL interaction with *MHO1*. We had a closer look downstream of IP3, the precursor to IP4, IP5 and IP6. These phospholipids control many processes in yeast cells, such as nuclear mRNA export [21] and chromatin remodeling [22]. (For a full list of activities see Figure S5). To examine the involvement of this pathway in the SL phenotype, we tested if *MHO1* is SL with *ARG82/IPK2, IPK1, KCS1* or *VIP1*, the four inositol polyphosphate kinases downstream of Plc1. All of the double-deleted strains were viable and grew similarly to *memoΔ* cells, suggesting that none of the other InsPs are involved in the SL phenotype with *MHO1*. Thus, we can only speculate that IP3 has an activity outside of its role as a precursor to the other InsPs and that this is required for proliferation in the *memoΔ* cells.

The SL phenotype uncovered in yeast is particularly intriguing since one of the mammalian PLC isoforms, PLCγ1, was identified in the same screen that uncovered Memo; PLCγ1 associated with a different ErbB2 autophosphorylation site [1]. Individual knock-down of either Memo or PLCγ1 impaired directed cell motility and simultaneous KD of both proteins totally blocked migration [2]. Since the *memoΔplc1Δ* strain failed to proliferate, it was not possible to test for effects on migration. However, both proteins do have roles in invasion/migration. Plc1p is essential for the activity of a nitrogen-controlled signaling pathway that controls pseudohyphal growth, and as we show here, Mho1 overexpression blocks haploid invasive growth.

The fact that Memo is structurally homologous to a bacterial class of dioxygenases, makes it tempting to speculate that Memo might have enzymatic activity. However, as mentioned in the Introduction, it is unlikely to have the same activity as the bacterial LigB family. Our current working hypothesis is that Memo is an enzyme, which might have acquired additional cellular activities during evolution, one being to serve an adaptor function. Indeed, in SKBR3 breast tumor cells, it was recently shown that Memo controls the association of RhoA and its effector mDia with the plasma membrane in response to ErbB2 activation, thereby impacting on recruitment of actin-binding proteins, MT dynamics [23] and migration [2]. These results suggest that Memo might be acting as an adaptor to localize proteins (and their activity) to specific cellular sites in order to initiate biological responses, in this case motility. The fact that Memo is also present in the nucleus, suggests that it might also have activities in this compartment. Future work will be aimed at uncovering the other roles of Memo and identifying its putative enzymatic activity.

## Materials and Methods

### Strains and media

For a complete list and genotypes of yeast strains used in this work, see Table 1 and Table 2. For experiments with *S. cerevisiae* most strains are congenic with BY4741 BY4742, and BY4743 wild-type strains. The haploid and diploid bar-coded systematic deletion collections for nonessential genes are in the strains BY4741 and BY4742 and BY4743, respectively. These were purchased from Invitrogen™. For filamentous growth and invasion assays the yeast strain Σ1278B was used (kindly provided by P. Jenö, Biocenter, Basel). All deletion strains were made by PCR mediated homologous recombination using the *kanMX* [24] or the *natMX* [25] selection. Transformation of the yeast strains was done using the LiAc/SS Carrier DNA/PEG method as described [26]. The *A. gossypii* strains were constructed by PCR-based gene targeting as described [11] and were cultured as described [27]. Actin staining with Alexa Fluor 488 (Molecular Probes, Eugene, OR) was done as described [28].

**Table 1:** *S. cerevisiae* strains used in this study.

Name	Genotype	Reference
BY4741	*MATa; his3Δ1; leu2Δ0; met15Δ0; ura3Δ0*	[5]
BY4742	*MATα; his3Δ1; leu2Δ0; lys2Δ0; ura3Δ0*	[5]
BY4743	*MATa/MATα his3Δ0/his3Δ0; leu2Δ0/leu2Δ0; met15Δ0/MET15; LYS2/lys2Δ0; ura3Δ0/ura3Δ0*	[5]
DHY194	*MATα; ura3–52Δ1::GFP-TUB1-URA3(pAFS125); trp1Δ63; leu2Δ1; his3Δ200*	[31]
Y5563	*MATα; lyp1Δ; his3Δ1; leu2Δ0; ura3Δ0; met15Δ0; can1::Mfapr-His3*	[32]
Σ1278	*MATa; ura3Δ0*	[18]
ISY42	*MATα; his3Δ;1 leu2Δ0; lys2Δ0; ura3Δ0; mho1::natMX*	this study
ISY34	*MATa; his3Δ1; leu2Δ0; met15Δ0; ura3Δ0; plc1::kanMX*	this study
ISY5	*MATα; ura3–52Δ1::GFP-TUB1-URA3(pAFS125); trp1Δ63; leu2Δ1; his3Δ200 memoΔ1::klTRP1*	
ISY120	*MATa; ura3Δ0; mho1::natMX*	this study
ISY229	*MATa; ura3Δ0; mho1::URA3*	this study
ISY231	*MATa; ura3Δ0::BYintURA_PGAL_ADHterm_URA3*	this study
ISY129	*MATa; ura3Δ0::BYintURA_PGAL_6His-MHO1_ADHterm_URA3*	this study
ISY38	*MATα; lyp1Δ; his3Δ1; leu2Δ0; ura3Δ0; met15Δ0; can1::Mfapr-His3; mho1::natMX*	this study
ISY120	*MATa; ura3Δ0::mho1::natMX*	this study
ISY229	*MATa; ura3Δ0::mho1::URA3*	this study
ISY211	*MATa; ura3Δ0; pRS416*	this study
ISY203	*MATa; ura3Δ0; pRS416_pGAL_6HIS-MHO1*	this study
ISY203	*MATa; ura3Δ0; pRS416-6HIS-humanMEMO*	this study

**Table 2:** *A. gossypii* strains used in this study.

Name	Genotype	Reference
WT	*leu2Δ; thr4Δ* (*ΔlΔt*)	[33]
*AGR321WΔ*	*leu2Δ; thr4Δ; agr321w::GEN3*	this study

### DNA manipulations, plasmids and strain constructions

We applied a PCR-based method to construct gene deletion cassettes that were used in yeast transformations [24]. All deletion strains were made by using the *kanMX* [24], the *natMX* [25], the *URA3MX* [29] or the *klTRP1* [30] deletion cassettes. Transformation of the yeast strains was done using the LiAc/SS Carrier DNA/PEG method as described [26]. Correct genomic integration of the corresponding construct was verified by analytical PCR [24]. Yeast strains were grown on: YPD-G418 (200 mg/l geneticin) to select for transformants that had integrated *kanMX* deletion cassettes; or on YPD-CloNat (100 mg/l nourseothricin) to select for transformants that had integrated *natMX* deletion cassettes. To replace the yeast gene with the human gene, the *humanMEMO-natMX* cassette was integrated. Growth on SD plates lacking uracil or tryptophan selected for transformants that had integrated *URA3MX* deletion cassettes, selected for the integration of the uptake of the plasmid pRS416, or klTRP1 integration. Yeast strains were grown on YPD-5-FOA plates (100 mg/l 5-Fluoroorotic Acid) to select cells have lacking the *URA3* expressing pRS416 plasmid. The plasmids used in this work are listed in Table 3.

**Table 3:** Plasmids used in this study.

Plasmid	Reference:
pFA6_kanMX6	[24]
pFA_GFP(s65t)_kanMX6	[13]
pAG25	[25]
pRS416	[34]
pGEN3	[11]
pSO142	[35]
pRS416_pPLC1_PLC1_PLC1term	this study
pRS416_pGAL_ADHterm	this study
pRS416_pGAL_6HIS-MHO1_ADHterm	this study
BYintURA_pGAL_ADHterm	personal communication
BYintURA_pGAL_eGFP-hMEMO_ADHterm	this study
BYintURA_pGAL_MHO1_ADHterm	this study
pAG25-hMEMO	this study
pAG25_MYC-hMEMO	this study

### Generation of mouse embryonic fibroblasts (MEFs)

Mouse embryonic fibroblasts (MEFs) were generated by standard procedures from Memo fl/fl embryos and were spontaneously immortalized by continued passaging in Dulbecco's modified Eagle's medium (DMEM) supplemented with 10% Fetal Calf Serum (GIBCO Invitrogen AG, Basel, Switzerland) and Penicillin and Streptomycin (growth medium).

### Microscopy

For yeast microscopy we used an Axioimager Z1 microscope (Carl Zeiss, Feldbach, Switzerland), equipped with a X-Cite 120 EXFO Metal Halide for fluorescence and Halogen for TL, a motorized XYZ stage, and a Plan-APOCHROMAT 100×/1.4 DIC Oil objective. GFP/Alexa488 (Zeiss #10) and DAPI (Zeiss #49) filter sets were used (Carl Zeiss, Feldbach, Switzerland). Fluorescence excitation was controlled by a shutter controller. We used a AxioCam MRm (1388×1040 pixels, Pixel size 6.45 µm) back-illuminated, cooled charge-coupled device camera mounted on the primary port. We used the AxioVision software from Zeiss for data acquisition, processing and analysis.

SKBR3 (ATCC, Manassas, Virginia) cells were grown on glass coverslips (BD Biosciences, San Diego, CA) coated with 25 µg/ml rat-tail collagen I (Roche Diagnostics) and MEFs were grown on μ-Slides 8well from ibidi (Martinsried, Germany), in DMEM containing 10% FCS at 37°C and stimulated with 10 nM HRG for 20 minutes. Cells were fixed with 4% paraformaldehyde and 3% sucrose in PBS, permeabilized in 0.2% Triton X-100 in PBS, and blocked with 1% BSA in PBS before incubation with the primary rat anti-α-tubulin antibody and mouse anti-Memo antibody. Alexa-Fluor 546 conjugated anti-rat antibody (Molecular Probes, Eugene, OR) and Alexa-Fluor 488 conjugated anti-mouse antibody (Molecular Probes, Eugene, OR) were used as secondary antibodies. F-actin was stained at room temperature with 2 ^U^/_ml_ FITC-labeled phalloidin (Molecular Probes, Eugene, OR). Cells were washed with PBS-Tween 0.1% and mounted with a mounting solution (ProLong® Gold Antifade Reagent, Molecular Probes). Mounted samples were examined using Axioimager Z1 microscope (Carl Zeiss, Feldbach, Switzerland) or using the Laser Scanning Microscope Axio Imager Z2 with the LSM 700 scanning head.

Pictures from MEFs were taken with the following equipment: The Axioimager Z1 microscope (Carl Zeiss, Feldbach, Switzerland) was equipped with a X-Cite 120 EXFO Metal Halide for fluorescence and Halogen for TL, a motorized XYZ stage, and a Plan-APOCHROMAT 100×/1.4 DIC Oil objective. GFP/Alexa488 (Zeiss #10) and DAPI (Zeiss #49) filter sets were used (Carl Zeiss, Feldbach, Switzerland). Fluorescence excitation was controlled by a shutter. We used a AxioCam MRm (1388×1040 pixels, Pixel size 6.45 µm) back-illuminated cooled charge-coupled device camera mounted on the primary port. We used the AxioVision software from Zeiss for data acquisition, processing and analysis.

Pictures from SKBR3 cells were taken with the following equipment: Axio Imager Z2 with the LSM 700 scanning head was equipped with the Laser lines: 405 (5 mW), 488 (10 mW), 555 (10 mW), 639 (5 mW); and the Lights: Coolled 405, 490, 565 (RL), Halogen (TL), a motorized XYZ stage, and the objectives: Plan-Apochromat 20×/0,8 M27 EC Plan-Neofluar 40×/1,30 Oil M27 Plan-Apochromat 63×/1,40 Oil DIC M27. The Axio Imager Z2 had 2 Epifluorescence PMTs and 1 Transmission PMT. For acquisition we used the ZEN2010 software from Zeiss. Data were further processed and analyzed with Imaris from Bitplane Scientific Software (Zurich, Switzerland).

### Signal intensity quantification

Z-stack Images taken with the Axio Imager Z2 with the LSM 700 scanning head were analyzed with ZEN2010 software and 3D image reconstruction was processed with IMARIS software (Bitplane AG, Zurich, Switzerland). In order to determine the nuclear intensity of the Memo staining, nuclear surfaces were created using the automatic threshold and pixels from this area were extracted from the total surface area to create the cytoplasmic surface. The mean intensities from these two surfaces were then measured. The mean differences in starved cells were compared to those in treated cells using the Student's t-test.

### Invasion assay

Wild-type, *mho1Δ* and *MHO1* overexpressing Σ1278b strains were streaked out on YPD (yeast extract/peptone/dextrose) and YPGal (yeast extract/peptone/galactose) plates and were grown for 4–5 days at 30°C. The plates were washed under a constant stream of tap water to wash off non-invasive strains.

### SL screen

In the yeast strain haploid matingtype-selectable Y5563 (*MATα; lyp1Δ; his3Δ1; leu2Δ0; ura3Δ0; met15Δ0*; *can1::Mfapr-His3*) strain we deleted *MHO1* with the *natMX* deletion cassette and thus making it resistant to nourseothricin. The Y5563 *mho1::natMX* strain was then subsequently mated with each of the 4800 viable haploid deletion strains that were purchased from invitrogen™. The mating was performed on agar plates in a 96-well format using standard methods on a singer pinning robot (RoToR HDA pinning robots from singer; www.singerinstruments.com).

## Supporting Information

Figure S1 **Spotting assay of wild-type and** ***mho1Δ*** **strains on various compounds.** A wild-type and a *mho1Δ* strain were serial diluted and spotted on YPD plates (A) or SD complete plates (B), containing 800 µM CoCl_2_, 100 µM benomyl, or 100 µM rapamycin (A and B) and 800 µM NaCl, or HU 100 µM (B). No differences in growth between the *mho1Δ* and the wild-type strains were observed.
(TIF)

Figure S2 ***MHO1*** **expression analysis.** A summary of the published *MHO1* microarray data is presented. Conditions that increase or decrease MHO1 expression are indicated in red or green, respectively. The data were taken from http://transcriptome.ens.fr/ymgv/. *MHO1* is upregulated upon Msn2 overexpression leading to the hypothesis that MHO1 is a stress response gene.
(TIF)

Figure S3 **Expression levels of** ***MHO1*** **and** ***PLC1*** **in response to different stress conditions.** Microarray data from [19] analyzing the gene expression of yeast cells in response to environmental stress was used to identify conditions that increase expression levels of *MHO1*. The *PLC1* expression levels in response to the same conditions are also shown.
(TIF)

Figure S4 ***MHO1*** **promoter analysis.** (A) An analysis of the MHO1 promoter region (−1 bp to −500 bp from the START codon) revealed that there are two potential binding sites for Msn2/Msn4 (shown in green) and a potential UAS_ino_ (inositol-sensitive upstream activating sequence) binding site. The latter is often present in promoters of genes encoding phospholipid, fatty acid, and sterol biosynthetic enzymes. (B) Using the YEASTRACT (Yeast Search for Transcriptional Regulators And Consensus Tracking; database (http://www.yeastract.com/index.php), we identified a list of transcription factors that can potentially directly or indirectly regulate *MHO1* transcription.
(TIF)

Figure S5 **IP_3_ signaling pathway.** IP_3_ and DAG are produced by the cleavage of PIP_2_ by Plc1. IP_3_ is released from the membrane and is the precursor of all other inositol phosphates (IPs). The four inositol polyphosphate kinases (Ipk2/Arg82, Ipk1, Ksc1, and VIP1) further process IP_3_ and constitute a nuclear signaling pathway. The major functions affected by the different IPs and the primary references are shown in this figure. IP_4_→Gene expression and Chromatin remodelling: [36], [37], [38], [39], [40], [41]. IP_5_→Telomere length and DNA repair: [42], [43], [44], [45]. IP_6_→mRNA Export, Non Homologous End Joining, RNA editing: [21], [46], [47], [48], [49].
(TIF)

Table S1 Genes involved in the cAMP/PKA/PLC pathway, which were tested with the *mho1Δ* strain; none were SL with *mho1Δ*.
(DOCX)
