# Supplementary figures and images for: MHO1, an Evolutionarily Conserved Gene, Is Synthetic Lethal with PLC1; Mho1p Has a Role in Invasive Growth
Source: PLoS One. 2012 Mar 7;7(3):e32501. doi: 10.1371/journal.pone.0032501 (PMC3296727; doi:10.1371/journal.pone.0032501)

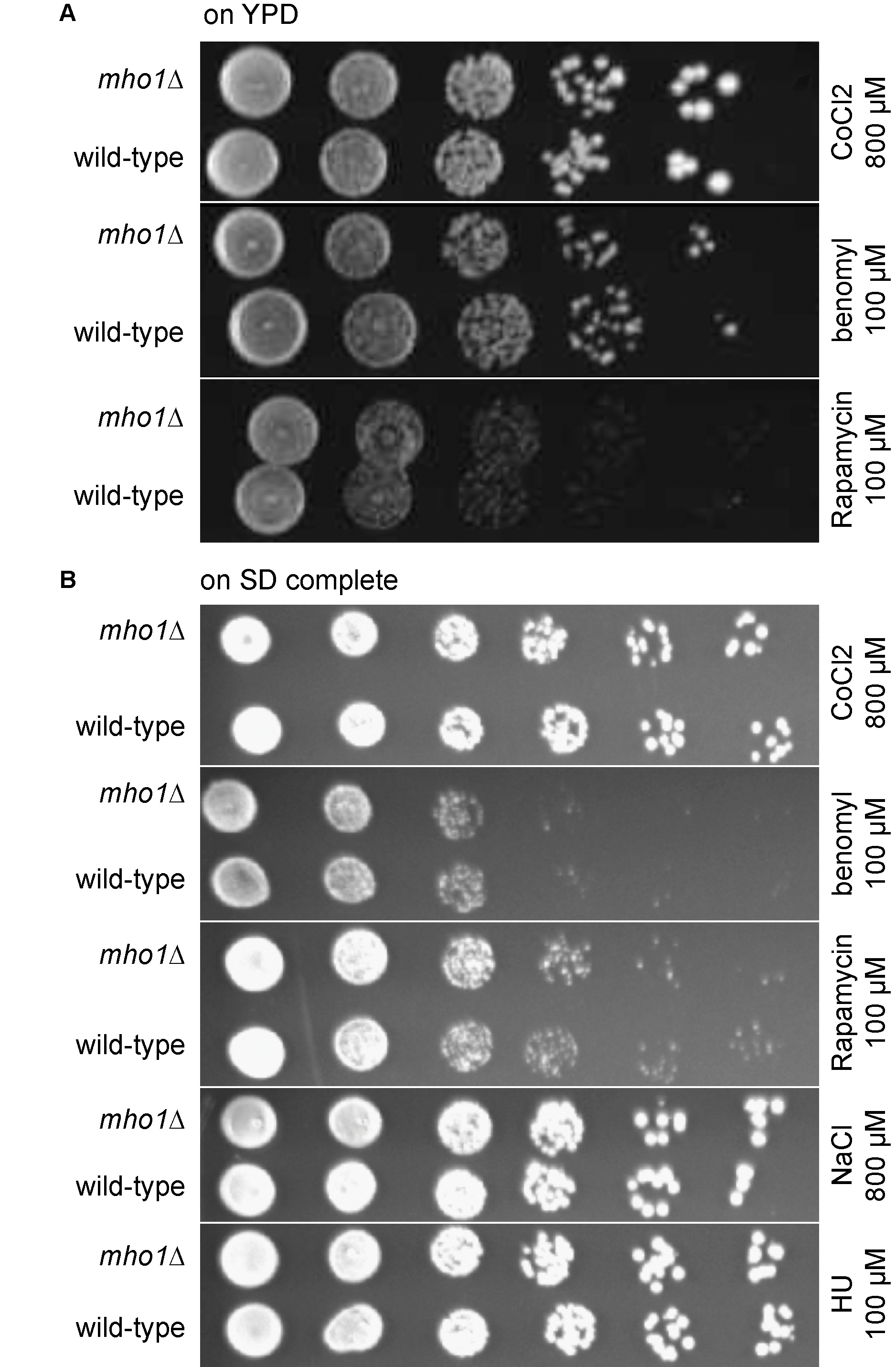

Supplement: Figure S1 — Spotting assay of wild-type and mho1Δ strains on various compounds. A wild-type and a mho1Δ strain were serial diluted and spotted on YPD plates (A) or SD complete plates (B), containing 800 µM CoCl2, 100 µM benomyl, or 100 µM rapamycin (A and B) and 800 µM NaCl, or HU 100 µM (B). No differences in growth between the mho1Δ and the wild-type strains were observed. (TIF) [file pone.0032501.s001.tif]

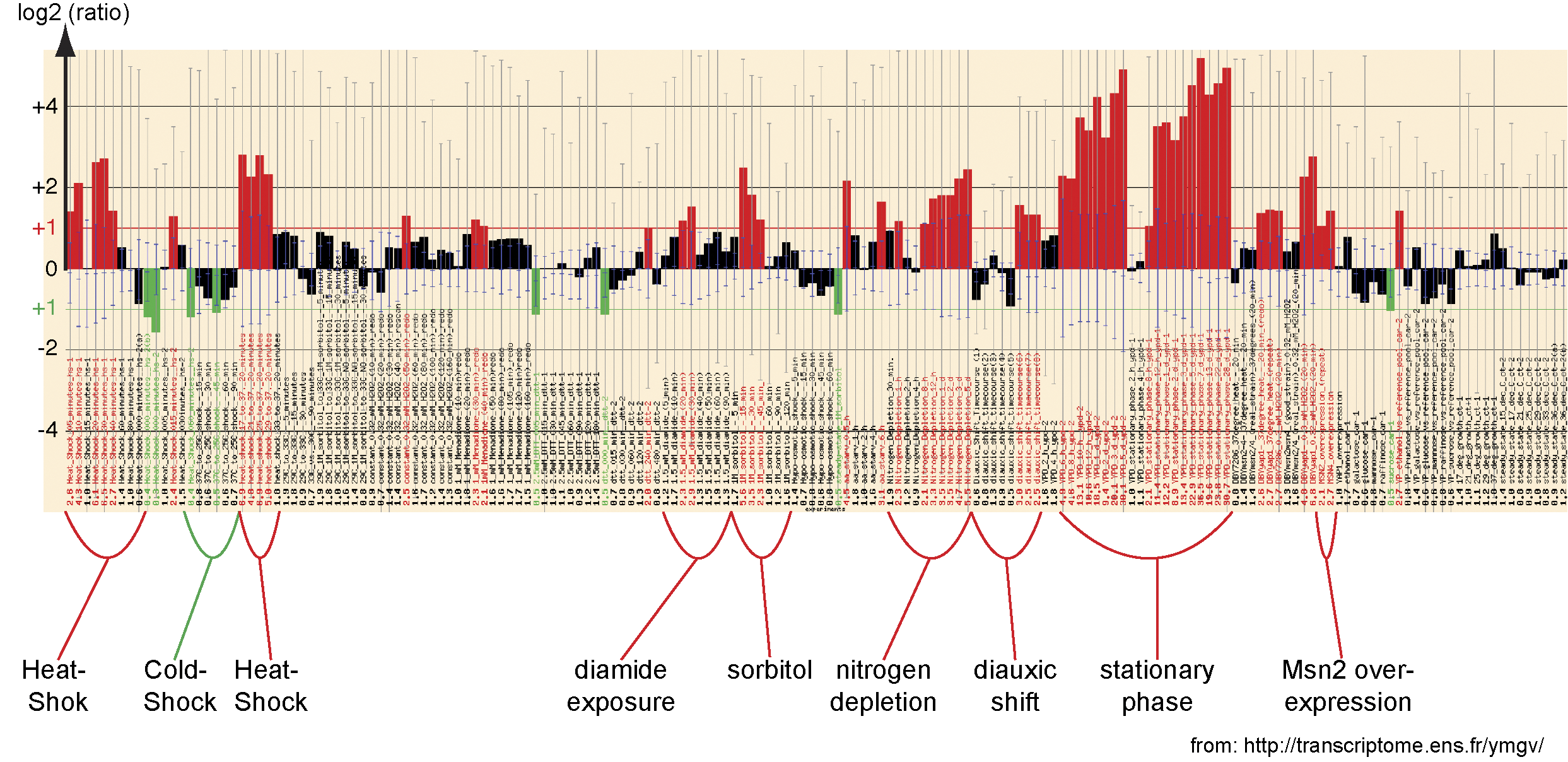

Supplement: Figure S2 — MHO1 expression analysis. A summary of the published MHO1 microarray data is presented. Conditions that increase or decrease MHO1 expression are indicated in red or green, respectively. The data were taken from http://transcriptome.ens.fr/ymgv/. MHO1 is upregulated upon Msn2 overexpression leading to the hypothesis that MHO1 is a stress response gene. (TIF) [file pone.0032501.s002.tif]

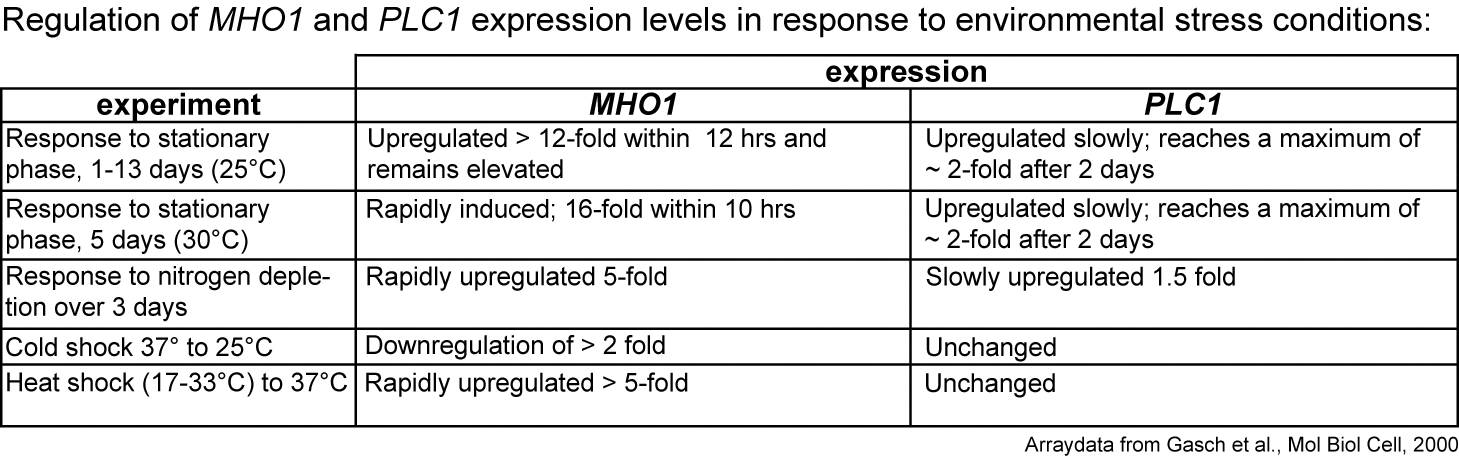

Supplement: Figure S3 — Expression levels of MHO1 and PLC1 in response to different stress conditions. Microarray data from [19] analyzing the gene expression of yeast cells in response to environmental stress was used to identify conditions that increase expression levels of MHO1. The PLC1 expression levels in response to the same conditions are also shown. (TIF) [file pone.0032501.s003.tif]

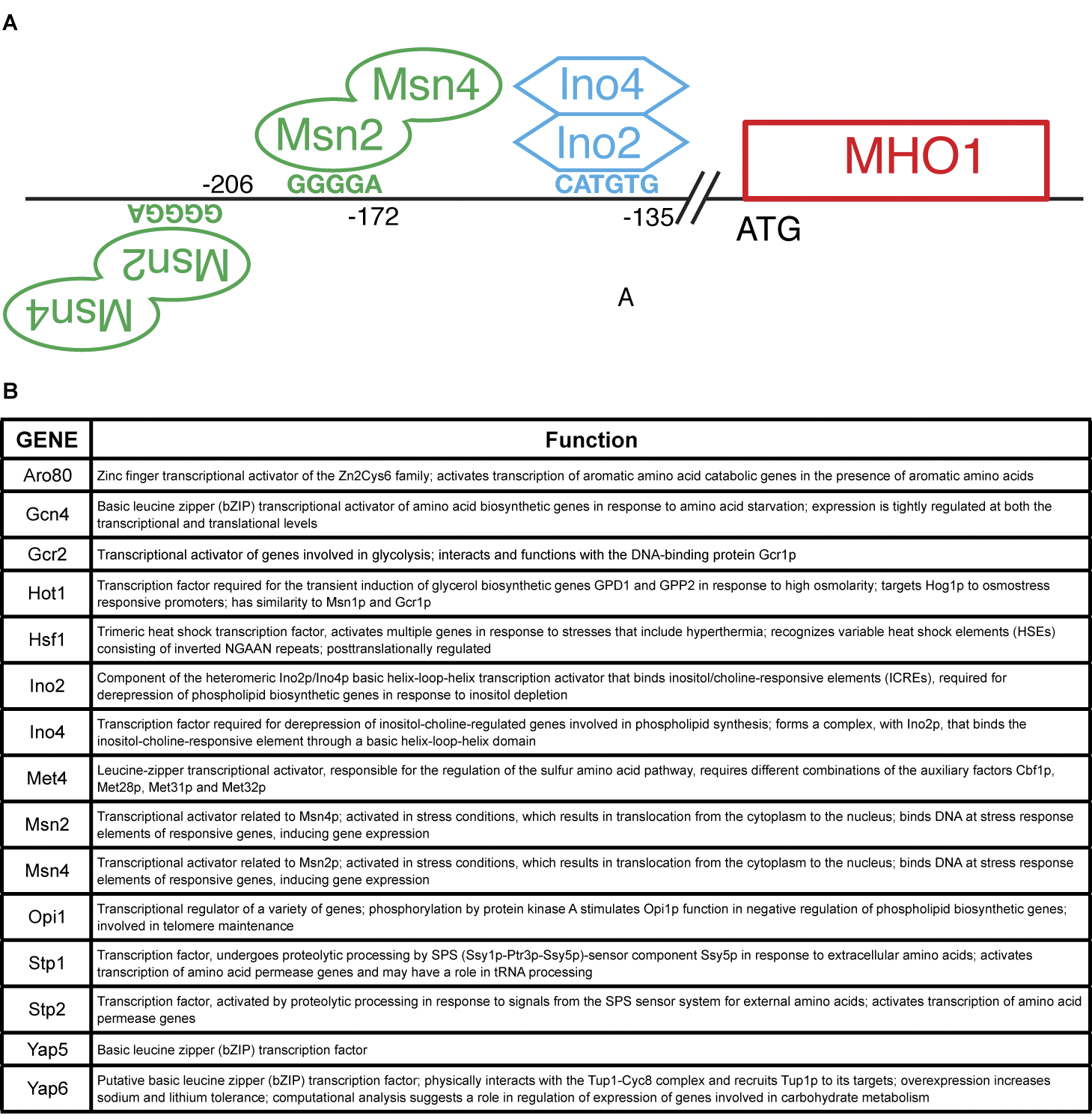

Supplement: Figure S4 — MHO1 promoter analysis. (A) An analysis of the MHO1 promoter region (−1 bp to −500 bp from the START codon) revealed that there are two potential binding sites for Msn2/Msn4 (shown in green) and a potential UASino (inositol-sensitive upstream activating sequence) binding site. The latter is often present in promoters of genes encoding phospholipid, fatty acid, and sterol biosynthetic enzymes. (B) Using the YEASTRACT (Yeast Search for Transcriptional Regulators And Consensus Tracking; database (http://www.yeastract.com/index.php), we identified a list of transcription factors that can potentially directly or indirectly regulate MHO1 transcription. (TIF) [file pone.0032501.s004.tif]

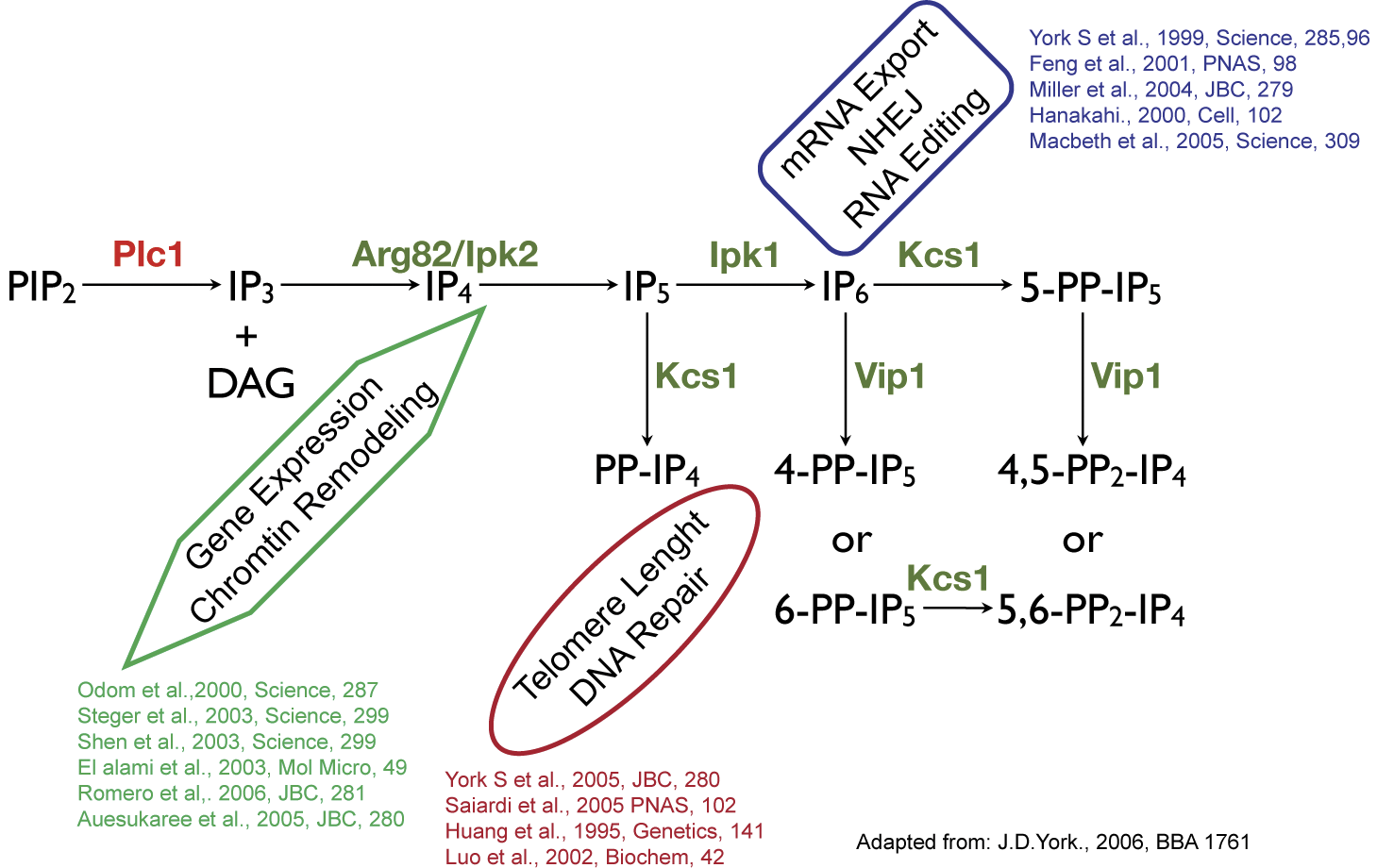

Supplement: Figure S5 — IP3 signaling pathway. IP3 and DAG are produced by the cleavage of PIP2 by Plc1. IP3 is released from the membrane and is the precursor of all other inositol phosphates (IPs). The four inositol polyphosphate kinases (Ipk2/Arg82, Ipk1, Ksc1, and VIP1) further process IP3 and constitute a nuclear signaling pathway. The major functions affected by the different IPs and the primary references are shown in this figure. IP4→Gene expression and Chromatin remodelling: [36], [37], [38], [39], [40], [41]. IP5→Telomere length and DNA repair: [42], [43], [44], [45]. IP6→mRNA Export, Non Homologous End Joining, RNA editing: [21], [46], [47], [48], [49]. (TIF) [file pone.0032501.s005.tif]
