# Supplementary material for: MHO1, an Evolutionarily Conserved Gene, Is Synthetic Lethal with PLC1; Mho1p Has a Role in Invasive Growth
Source: PLoS One. 2012 Mar 7;7(3):e32501. doi: 10.1371/journal.pone.0032501 (PMC3296727; doi:10.1371/journal.pone.0032501)
Supplement: Table S1 — Genes involved in the cAMP/PKA/PLC pathway, which were tested with the mho1Δ strain; none were SL with mho1Δ. (DOCX) [file pone.0032501.s006.docx]

**Supplemental Table 1:** Genes involved in the cAMP/PKA/PLC pathway, which were tested with the *mho1Δ* strain*;* none were SL with *mho1Δ*.

| **GENE** | **FUNCTION (www.yeastgenome.org)** |
| --- | --- |
| BUB1 ^1^ | Protein kinase involved in the cell cycle checkpoint into anaphase; forms complex with Mad1p and Bub3p crucial to preventing cell cycle progression into anaphase in the presence of spindle damage |
| BUB3 ^1^ | Kinetochore checkpoint WD40 repeat protein that localizes to kinetochores during prophase and metaphase, delays anaphase in the presence of unattached kinetochores |
| CBF1 ^1^ | Dual function helix-loop-helix protein; binds the motif CACRTG present at centromere DNA element I (CDEI); affects nucleosome positioning at this motif; associates with kinetochore proteins and required for efficient chromosome segregation |
| MAD2 ^1^ | Component of the spindle-assembly checkpoint complex; delays the onset of anaphase in cells with defects in mitotic spindle assembly; regulates APC/C activity during prometaphase and metaphase of meiosis I |
| ARG82/IPK2 ^2^ | Inositol polyphosphate multikinase (IPMK), sequentially phosphorylates Ins(1,4,5)P3 to form Ins(1,3,4,5,6)P5; also has diphosphoinositol polyphosphate synthase activity; regulates arginine-, phosphate-, and nitrogen-responsive genes |
| IPK1 ^2^ | Inositol 1,3,4,5,6-pentakisphosphate 2-kinase, nuclear protein required for synthesis of 1,2,3,4,5,6-hexakisphosphate (phytate), which is integral to cell function |
| KCS1 ^2^ | Inositol hexakisphosphate (IP6) and inositol heptakisphosphate (IP7) kinase; generation of high energy inositol pyrophosphates by Kcs1p is required for many processes such as vacuolar biogenesis, stress response and telomere maintenance |
| VIP1 ^2^ | Inositol hexakisphosphate (IP6) and inositol heptakisphosphate (IP7) kinase; IP7 production is important for phosphate signaling; involved in cortical actin cytoskeleton function, and invasive pseudohyphal growth |
| RAS1 ^3^ | GTPase involved in G-protein signaling in the adenylate cyclase activating pathway, plays a role in cell proliferation; localized to the plasma membrane; homolog of mammalian RAS proto-oncogenes |
| RAS2 ^3^ | GTP-binding protein that regulates the nitrogen starvation response, sporulation, and filamentous growth; farnesylation and palmitoylation required for activity and localization to plasma membrane; homolog of mammalian Ras proto-oncogenes |
| IRA2 ^3^ | GTPase-activating protein that negatively regulates RAS by converting it from the GTP- to the GDP-bound inactive form, required for reducing cAMP levels under nutrient limiting conditions, has similarity to Ira1p and human neurofibromin |
| GPA2 ^3^ | Nucleotide binding alpha subunit of the heterotrimeric G protein that interacts with the receptor Gpr1p, has signaling role in response to nutrients; green fluorescent protein (GFP)-fusion protein localizes to the cell periphery |

^1^: Published genes having a SL phenotype with *plc1Δ*

^2^: The four inositol polyphosphate kinases downstream of Plc1 which further process IP_3_ to IP_4_, IP_5_, and IP_6_.

^3^: Genes involved the cAMP/PKA pathway signalling
